# A research agenda for digital payments of health workers in large-scale health campaigns in sub-Saharan Africa

**DOI:** 10.1136/bmjgh-2024-017476

**Published:** 2026-02-15

**Authors:** Peter Waiswa, Juliet Aweko, Charles Opio, Maggie Ssekitto Ashaba, Uchenna Igbokwe, Eric Aigbogun, Zahra Mboup, Souleymane Ndiaye, Adama Faye, Andrew Bakainaga, Elizabeth Ekirapa Kiracho

**Affiliations:** 1 Health Policy, Planning and Management, Makerere University College of Health Sciences, Kampala, Central Region, Uganda; 2 Global Public Health, Karolinska Institute, Stockholm, Sweden; 3 Solina Centre for International Development and Research, Abuja, Nigeria; 4 Universite Cheikh Anta Diop, Dakar, Senegal; 5 World Health Organization, Kampala, Uganda

**Keywords:** Global Health, Child health, Health systems, Immunisation, Public Health

## Abstract

**Introduction:**

Digital payments are increasingly favoured over cash for remunerating healthcare workers in large-scale health campaigns due to perceived advantages in efficiency and security. However, evidence to guide their scaling and optimisation is limited. This study aimed to identify and prioritise a global research agenda for digital payments in health campaigns in sub-Saharan Africa (SSA).

**Methods:**

We employed the Child Health and Nutrition Research Initiative methodology. In stage 1, we defined the context and criteria (answerability, feasibility, sustainability/equity, impact). In stage 2, 420 stakeholders were engaged via an online survey, generating 450 research questions, which were refined to a final pool of 35. In stage 3, these 35 questions were scored by 63 experts against the predefined criteria. Research Priority Scores (RPS) and Average Expert Agreement (AEA) were computed for ranking in stage 4.

**Results:**

The overall RPS for the 35 questions ranged from 38.6% to 6.0% (mean 28.2%, SD 6.4%). The AEA ranged from 67.2% to 82.7% (mean 77%, SD 3.4%), indicating strong consensus. RPS and AEA showed a strong positive correlation (r=0.989, p<0.01). The top-ranked research questions were: (1) Minimum requirements for health systems to digitise payments responsibly (RPS 38.6%); (2) Optimisation of digital payments to enhance campaign effectiveness in SSA (RPS 36.8%); (3) Incentives for digital payment adoption in the healthcare sector (RPS 36.1%); (4) Cost–benefit analysis of digital payments vs cash (RPS 36.3%) and (5) Coverage of mobile money agents and its impact on uptake and satisfaction (RPS 34.0%).

**Conclusions:**

This study provides an expert-consensus roadmap for research on digital payments in health campaigns. Addressing these priorities will generate critical evidence to develop robust, equitable and effective digital payment systems, ultimately strengthening health systems and improving health outcomes in SSA.

**WHAT IS ALREADY KNOWN ON THIS TOPIC**

- Cash payments in large-scale health campaigns are often slow, inefficient and insecure, leading to delays and demotivated health workers. Digital payments have been piloted with promising results, but a systematic evidence base to guide their scaling is lacking.

**WHAT THIS STUDY ADDS**

- This study uses a formal priority-setting method to establish an expert-consensus research agenda for digital payments in health. It identifies the top 35 questions, with the highest priorities being system requirements, optimisation, incentives and cost-effectiveness.

**HOW THIS STUDY MIGHT AFFECT RESEARCH, PRACTICE OR POLICY**

- This prioritised list provides a roadmap for researchers, funders and policymakers. Directing resources towards answering these questions will build the necessary evidence to implement effective, equitable and sustainable digital payment systems across sub-Saharan Africa.

## Introduction

 The Global Polio Eradication Initiative has made significant strides towards eradicating poliovirus worldwide.^1 2^ However, sustaining these achievements remains challenging. In Africa and Asia, cash-based payments for outbreak campaigns have caused delays, poor-quality operations and demotivated workers due to late payments.^1^ In the first quarter of 2020, 50% of polio outbreak campaigns in the African Region were delayed or adversely affected by slow fund distribution to the operational level.^1 2^ To address these challenges, the WHO Immunization Agenda 2030 (IA2030) identifies rapid fund transfers as critical for epidemic preparedness and swift outbreak responses.^1 3^

Pilot programmes implementing digital payments have shown promise in addressing these inefficiencies. For example, in Côte d'Ivoire, payment time for a campaign dropped from an average of 3 weeks for cash payments to just 2 hours using mobile money.^4^ Similarly, at least 50 000 front-line campaign workers in Côte d'Ivoire, Mali and Ghana were paid promptly and transparently via mobile money in 2020.^1 2 5^ Despite these promising pilots, there is limited systematic evidence to guide the scaling and optimisation of digital payment systems across diverse sub-Saharan African (SSA) contexts. This gap is particularly critical given that half of SSA’s population is unbanked, facing difficulties in accessing traditional banking services.^6^ Mobile money has emerged as a transformative solution, but its rapid evolution brings challenges including security vulnerabilities, interoperability issues and regulatory hurdles that must be addressed for sustainable growth.^4^

While regulatory frameworks for mobile money are evolving across SSA, progress is uneven.^4 7^ This variation underscores the need for context-specific solutions. Efforts are underway to scale up digital payment programmes globally to accelerate funding distribution and enhance worker satisfaction. However, a coherent and evidence-informed research agenda is lacking to guide this expansion. Previous research has been fragmented, focusing on isolated pilots or specific technical aspects, without a comprehensive prioritisation of the most critical evidence gaps. This study, therefore, aims to systematically identify and prioritise a global research agenda for digital payments in health campaigns in SSA, providing a roadmap for researchers, funders and policymakers.

## Methods

### Study design

We adopted the Child Health and Nutrition Research Initiative (CHNRI) methodology, a systematic, consultative approach for setting priorities in health research.^8^ The core objective of CHNRI is to create a transparent and democratic process for generating a consensus on research priorities within a specific field. While CHNRI can be operationalised through up to 15 detailed steps, its framework is flexible and often consolidated into four key stages: (1) context and criterion setting; (2) generating and refining research options; (3) scoring options and (4) data analysis and prioritisation.^8 9^ This consolidated, four-stage approach has been successfully used in numerous priority-setting exercises published in peer-reviewed literature.^10 11^

For this study, we adapted the standard CHNRI methodology. This adaptation follows the principle of broadening participation in the question-generation phase, an approach documented in methodological reviews of research prioritisation to mitigate the risk of overlooking critical issues framed by front-line practitioners and regionally based experts.^12^ We broadened this first stage by also soliciting questions directly from a large pool of experts via an online survey to enhance inclusivity and capture a wider range of perspectives. A noted strength of such an approach is the increased diversity of input; a potential limitation is the increased complexity of synthesising a large volume of suggestions into a coherent list, which we mitigated through a structured review by the Digital Health Payment Initiative and Research (DHPIR) secretariat based at the Makerere University of Public Health.

**Figure 1:**
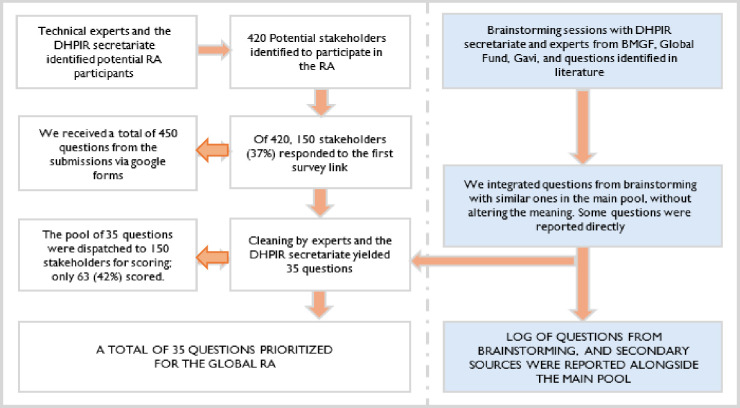
Flow chart showing the steps in the development of the Research Agenda (RA). The flow chart depicts the four-stage CHNRI methodology. It begins with the identification of 420 potential stakeholders from technical experts and the DHPIR secretariat. In stage 2 (question generation), 150 stakeholders (36%) responded to the survey, submitting 450 raw research questions. These were combined with questions from brainstorming sessions with several teams including the Bill & Melinda Gates Foundation (BMGF). The DHPIR secretariat and technical experts cleaned and synthesised this pool, removing duplicates and rephrasing for clarity, resulting in a final list of 35 distinct questions. In stage 3 (scoring), these 35 questions were dispatched to the same 150 stakeholders for evaluation; 63 stakeholders (42%) completed the scoring. Stage involved data analysis to generate the final prioritised research agenda. CHNRI, Child Health and Nutrition Research Initiative; DHPIR, Digital Health Payment Initiative and Research.

The rationale for this adaptation was to ensure the research agenda was grounded in the diverse, real-world experiences of a larger and more varied expert community. The process consisted of four stages, as detailed below and summarised in figure 1.

#### Stage 1: Defining context, domains and criteria

The study context was defined as digital payments for remunerating health workers in large-scale campaigns (eg, vaccination, mass drug administration) in SSA. We identified experts and stakeholders from programme, research, donor, government and policy backgrounds through online databases (PubMed, Google Scholar, HINARI), snowballing techniques and recommendations from institutions and governments. The prioritisation criteria were defined as: Answerability (the likelihood a question can be answered by a well-designed study), Feasibility (the practicality of conducting the research), Sustainability and Equity (the potential for the research to lead to sustainable and equitable outcomes) and Impact (the potential for the research to have a significant effect on policy or practice).

#### Stage 2: Formulating and refining research questions

An initial long-list of research questions was generated from two primary sources: (1) brainstorming meetings with key stakeholders (eg, from the Global Fund, Gavi, Bill & Melinda Gates Foundation, Better Than Cash Alliance) and (2) an online survey disseminated to 420 identified experts, who were asked to submit at least two research questions each. Out of the 420 stakeholders contacted, 150 voluntarily participated in this phase (response rate: 36%), submitting a total of 450 research questions. The DHPIR secretariat, in consultation with technical experts, then reviewed and refined this pool. The process involved removing duplicates, merging similar questions and rephrasing for clarity, resulting in a final, distinct list of 35 research questions for scoring. The questions from the brainstorming sessions are listed in online supplemental table 1.

It is important to note that this was a process of synthesising a long list of research ideas into a manageable set of distinct research questions for scoring, not the statistical development of a psychometric scale. Therefore, techniques like principal component analysis for item reduction were not appropriate. Instead, the refinement followed established CHNRI practices, where a technical working group synthesises and de-duplicates suggestions from experts to create a clean list for scoring, ensuring the final options are clear, comprehensive and non-overlapping.^9 12^ This resulted in a refined list of 35 distinct research questions categorised into four thematic areas: Efficiency and Effectiveness, Digital Payment Processes, Financial Inclusion and Economic Empowerment, and Adoption and Acceptance.

#### Stage 3: Scoring of questions

The refined list of 35 questions was sent for scoring to the same 150 stakeholders who had participated in the question-generation phase (stage 2), via an online Google Form. Respondents were asked to score each question against the four predefined criteria (Answerability, Feasibility, Sustainability/Equity, Impact) using a 5-point Likert scale (1=Very Low to 5=Very High).

#### Stage 4: Data management, analysis and prioritisation

Data from completed forms were analysed in STATA V.15. We computed two main metrics:

Research Priority Score (RPS): This indicates the ‘collective optimism’ that a research question satisfies all evaluation criteria.^9^ RPS was computed by: (1) normalising the Likert scores (dividing by 5); (2) calculating the average of the normalised scores across all experts for each criterion (intermediate scores) and (3) calculating the RPS for each question by taking the product of the intermediate scores across all four criteria. The RPS inherently accounts for the collective judgement on all criteria simultaneously. While it does not explicitly model the variability of scores for each question, the high Average Expert Agreement (AEA) values reported below indicate that this variability was low, supporting the robustness of the mean scores used in the RPS calculation.

AEA: This assesses the level of consensus among experts when scoring questions. AEA was computed by calculating the proportion of pairs of experts who gave the same score for each question and then averaging these pairwise agreements across all questions. It is acknowledged that AEA does not calculate agreement beyond chance, which a kappa statistic would. However, AEA is a commonly reported and accepted measure of consensus in CHNRI exercises, providing a transparent indicator of the degree of alignment among experts.^9 13^ The research questions were ranked in descending order of their RPS, with higher scores representing higher priority.

### Patient and public involvement

Neither patients nor the public were involved in the design, conduct, reporting or dissemination plans of this research.

## Results

### Participant characteristics

Of the 420 stakeholders contacted for participation, 150 (36%) responded and voluntarily submitted questions in the generation phase (stage 2), yielding a total of 450 raw research questions. In the scoring phase (stage 3), the 35 refined questions were sent to these 150 participants for evaluation; 63 out of the 150 completed the scoring survey (response rate: 42% of stage 2 participants, 15% of the originally contacted pool). Most respondents were researchers identified via PubMed, academics from various universities, government officials from ministries of finance and health, and representatives from donor agencies.

### Research Priority Scores

The RPS for the 35 questions ranged from 38.6% to 6.0%, with a mean of 28.2% (SD=6.4) and a median of 28% (IQR, IQR=28.9%–31.5%) (online supplemental table 2). The top five ranked research questions are presented in online supplemental table 4. The majority of questions (19/35, 54%) fell under the ‘Efficiency and effectiveness of digital payment platforms’ theme. This theme also dominated the top 15 questions, accounting for 9 (60%) of them (online supplemental table 3). The ‘Digital payment processes’ theme contributed three questions (20%) to the top 15, while ‘Financial inclusion and economic empowerment’ contributed 2 (13%). Notably, no questions from the ‘Adoption and acceptance’ theme appeared in the top 15.

### Average Expert Agreement

The AEA ranged from 67.2% to 82.7%, with a mean of 77% (SD=3.4%) and a median of 78.0% (IQR=76.5%–79.4%) (online supplemental table 2), indicating a strong overall consensus among scorers. There was a very strong positive correlation between the RPS and AEA (r=0.989, p<0.01), meaning that questions ranked as higher priority also had higher levels of expert agreement on their scores. The top five questions by RPS were also the top five by AEA, with AEA values all above 80%.

## Discussion

This study successfully developed an expert-consensus global research agenda to guide the implementation of digital payments in large-scale health campaigns in SSA. The high AEA of 77% and the strong correlation with RPS indicate robust consensus on the identified priorities. Our findings underscore that while digital payments are being scaled, critical evidence gaps remain regarding their foundational requirements, optimisation and cost-effectiveness.

The top-ranked question on ‘minimum requirements for responsible digitisation’ reflects a critical consensus on the need for robust frameworks. This aligns with lessons from other digital health transformations, such as the roll-out of electronic medical records in other low-income and middle-income countries, where a lack of foundational standards led to interoperability challenges and system failures.^14^ Similarly, the emphasis on cost–benefit analysis (rank 4) echoes a recurring theme in global health financing; for instance, economic evaluations were pivotal in scaling up successful interventions during Gavi’s vaccine introduction programmes and need to be similarly applied to the payment systems that support them.^15^

The dominance of ‘Efficiency and Effectiveness’ themes in the top priorities suggests that experts are still seeking conclusive proof that digital payments not only speed up transactions but also tangibly improve campaign outcomes, such as coverage and quality. This is a prudent focus, as demonstrated by the COVID-19 response, where the rapid scale-up of digital tools sometimes outpaced the evidence of their effectiveness, leading to variable results.^16^

A significant finding is the absence of ‘Adoption and Acceptance’ questions from the top 15. This indicates a potential blind spot in the current research trajectory, prioritising technical and economic aspects over human-centred ones. This gap is critical, as evidenced by past failures in digital health initiatives where user resistance, often due to low digital literacy or cultural factors, undermined technological potential.^17^ Future research must intentionally explore these sociobehavioural determinants to ensure digital payments are not only efficient but also widely accepted and used.

Findings from published studies from within the DHPIR programme of research provide preliminary insights.^18^,^20^ The strong preference for digital payments over cash due to security and time savings, as seen in Uganda and Malawi, mirrors positive experiences from other mobile money applications, like conditional cash transfers.^20 21^ However, the persistent barriers, limited internet, agent liquidity and low digital literacy, disproportionately affecting women, highlight systemic weaknesses. These findings are consistent with challenges observed in other SSA digital finance and health programmes, emphasising that technological solutions are only as strong as the systems they operate within.^4^ The gender dynamics observed, where digital payments can empower women but also create household tension, underscore the need for a ‘gender-transformative’ approach, a lesson also emerging from women’s financial inclusion programmes beyond health.^20 22^

### Contextual variations and future scope

The agenda presented here is a regional consensus for SSA. We acknowledge the significant geographical and contextual variations across the continent, such as the advanced regulatory landscape in Kenya versus the nascent stages in the DRC.^4 7^ These differences may affect the generalisability of findings from research conducted in one context to another. Therefore, while this agenda identifies universal priority topics, we strongly recommend that future research be designed to capture context-specific factors. Comparative studies across different regulatory and infrastructural environments would be particularly valuable in generating transferable lessons. Furthermore, this study focused on large-scale campaigns. The applicability of these priorities to digital payment systems for routine health programmes (eg, routine immunisation, primary healthcare worker salaries) remains a critical area for future research to ensure comprehensive policy guidance for the entire health system.

### Strengths and limitations

A key strength of this study is the application of the systematic CHNRI approach, which minimises selection bias and enhances the credibility of the output. The inclusion of a wide range of experts from Anglophone and Francophone Africa, as well as global and local organisations, increased the robustness of the agenda. However, several limitations must be acknowledged. The online survey methodology resulted in response rates of 36% and 42% in stages 2 and 3, respectively. While common in expert elicitation surveys, non-response can introduce bias if non-respondents systematically differ from respondents. To mitigate this, we ensured a wide and diverse recruitment of experts. The AEA measure, while indicating strong consensus, does not account for chance agreement. Future studies could employ the kappa statistic for a more robust measure of concordance. Finally, the refinement of questions, while following established CHNRI practice, involved subjective judgement by the secretariat; however, this was a necessary step to synthesise a large volume of ideas into a scorable list.

## Conclusion and call to action

This prioritised agenda provides a direct roadmap. We issue a call to action for funders, policymakers and researchers to:

Invest in answering the top five questions on system requirements, optimisation, incentives, cost–benefit and agent coverage to build the foundational evidence for scaling.

Intentionally target the gap in ‘Adoption and Acceptance’ research to understand and address user perspectives, ensuring digital payments are equitable and inclusive.

Expand the scope of inquiry to include context-comparative analyses and the role of digital payments in routine health system strengthening, not just campaigns.

Addressing these priorities will ensure that the transition to digital payments is evidence-based, leading to systems that are not only more efficient but also more equitable and effective, ultimately strengthening health systems and improving health outcomes across SSA.

## Supplementary material

10.1136/bmjgh-2024-017476 online supplemental table 1

10.1136/bmjgh-2024-017476 online supplemental table 2

10.1136/bmjgh-2024-017476 online supplemental table 3

10.1136/bmjgh-2024-017476 online supplemental table 4

10.1136/bmjgh-2024-017476 online supplemental file 1

## Data Availability

Data are available on reasonable request.

## References

[1] Global Polio Eradication Initiative (2023). Global polio eradication initiative, annual report 2022.

[2] Global Polio Eradication Initiative (2021). Polio Eradication Strategy 2022-2026: Delivering on a Promise.

[3] Immunization Agenda 2030 Partners (2023). Immunization agenda 2030: A global strategy to leave no one
behind. Vaccine (Auckl).

[4] Ozili PK (2018). Impact of digital finance on financial inclusion and
stability. *Borsa Istanbul Review*.

[5] William J (2024). Mobile money: the economics of m-pesa. https://www.nber.org/system/files/working_papers/w16721/w16721.pdf.

[6] Bank W (2022). The Global Findex Database 2021: Financial Inclusion, Digital Payments, and
Resilience in the Age of COVID-19.

[7] Ahiabenu K (2022). A Comparative Study of the Design Frameworks of the Ghanaian and Nigerian
Central Banks’ Digital Currencies (CBDC). *FinTech*.

[8] Rudan I, Gibson JL, Ameratunga S (2008). Setting priorities in global child health research investments: guidelines
for implementation of CHNRI method. Croat Med J.

[9] Rudan I (2016). Setting health research priorities using the CHNRI method: IV. Key
conceptual advances. *J Glob Health*.

[10] Bermudez LG, Williamson K, Stark L (2018). Setting global research priorities for child protection in humanitarian
action: Results from an adapted CHNRI exercise. PLoS One.

[11] Irvine C, Armstrong A, Nagata JM (2018). Setting Global Research Priorities in Pediatric and Adolescent HIV Using
the Child Health and Nutrition Research Initiative (CHNRI) Methodology. J Acquir Immune Defic Syndr.

[12] Tomlinson M, Darmstadt GL, Yousafzai AK (2019). Global research priorities to accelerate programming to improve early
childhood development in the sustainable development era: a CHNRI
exercise. J Glob Health.

[13] Yoshida S (2016). Approaches, tools and methods used for setting priorities in health
research in the 21(st) century. J Glob Health.

[14] Williams F, Boren SA (2008). The role of the electronic medical record (EMR) in care delivery
development in developing countries: a systematic review. Inform Prim Care.

[15] Lee LA, Franzel L, Atwell J (2013). The estimated mortality impact of vaccinations forecast to be administered
during 2011-2020 in 73 countries supported by the GAVI Alliance. Vaccine (Auckl).

[16] Whitelaw S, Mamas MA, Topol E (2020). Applications of digital technology in COVID-19 pandemic planning and
response. Lancet Digit Health.

[17] Gagnon M-P, Nsangou É-R, Payne-Gagnon J (2014). Barriers and facilitators to implementing electronic prescription: a
systematic review of user groups’ perceptions. J Am Med Inform Assoc.

[18] Waiswa P, McConnell M, Aweko J (2025). The effect of supporting districts to operationalise digital payments for
vaccination campaign workers: a cluster randomised controlled trial during the 2022
polio vaccination campaign in Uganda. BMJ Glob Health.

[19] Bukuluki P, Ndira S, Aweko J (2025). A qualitative study on gender relations and digital payments: healthcare
workers’ experiences during polio vaccination campaigns in Uganda and
Malawi. BMJ Glob Health.

[20] Adeniji FP, Adewole D, Bello S (2025). Associations between digital financial services and health campaign
workers’ perceived satisfaction, motivation and performance in
Nigeria. BMJ Glob Health.

[21] Aker JC, Boumnijel R, McClelland A (2016). Payment Mechanisms and Antipoverty Programs: Evidence from a Mobile Money
Cash Transfer Experiment in Niger. Econ Dev Cult Change.

[22] Duvendack M, Mader P (2019). Impact of financial inclusion in low‐ and middle‐income
countries: A systematic review of reviews. *Campbell Systematic Reviews*.

