# Supplementary material for: A research agenda for digital payments of health workers in large-scale health campaigns in sub-Saharan Africa
Source: BMJ Glob Health. 2026 Feb 15;10(Suppl 4):e017476. doi: 10.1136/bmjgh-2024-017476 (PMC12962003; doi:10.1136/bmjgh-2024-017476)
Supplement: online supplemental table 1 [file bmjgh-10-Suppl_4-s001.docx]

# SUPPLEMENTARY TABLE 1: BRAINSTORMING IDEAS GENERATED WITH STAKEHOLDERS TO GUIDE DEVELOPMENT OF THE RESEARCH AGENDA

| FOCUS AREAS | BARRIERS | QUESTIONS |
| --- | --- | --- |
| 1. QUESTIONS FROM OUR MEETING WITH FUNDERS, GLOBAL FUND, AND GAVI IN GENEVA | | |
| - VERIFICATION | - Protracted verification processes causing delays in digital payments | - Does digitalization of health workers enhance timely payment, retention and impact gender. - How does digitization impact malaria, routine immunization services, campaign effectiveness, campaign quality, inclusiveness, equity cost/cost effectiveness. |
| - ORGANIZATION OF LARGE-SCALE HEALTH CAMPAIGNS | - Campaign managers implemented as emergencies with limited management capacity - Many Implementation partners working in silos | - Case studies on: How perceptions of program, MNOs…. Process |
| - POLITICAL COMMITMENT - POLICIES IN COUNTRIES - ACCESSIBILITY - FEES PLUS EXCHANGE RATES - CRIMINALIZED POP. KEY VULNERABLE/ MARGINALIZED. | - Limited political commitment |  |
|  |  | - Assessment of policies in countries and how those impact or facilitates beneficiaries in these settings. - What are alternatives to complement digital payment. - How best to educate beneficiaries around scheming, manipulation, fraud within their digital wallets. - Cost benefit analysis. - What can push people to use more compatible market rates. - How best to mitigate risk/ incentivize/ joining/ use/ receiving services? |
| - NEGATIVE EFFECTS WITHIN GENDER. | - Financial security. - Lack of relevant financial products to support the system. - Manual verification. - Gender violence. - Increase financial inclusion and improve financial behavior. - Economic empowerment, independent participatory planning. - Adequate cash at cash out points. - Inadequate resources. - Relevant policies. - Change management - Personal safety. - Lack of consolidated guidelines | - Evaluate these root causes plus test solutions. - Specific research - How cost effectiveness is digital payment - How can the timely payment of beneficiaries be promoted? - How does digital payment affect the performance of health workers programs? - What is the level of digital and financial literacy and how has digital payment affected the livelihood of beneficiaries? - What are the factors that are contributing to fast and slow adoption of digital payment? - How does conflict impact the use of digital payments and how can we mitigate the challenges? - How can we mitigate the gender related consequences that stem from digital payment? - How can we promote personal and financial safety and security? - What are the prerequisites for an effective digital payment system? |
| 1. QUESTIONS FROM ENGAGEMENT MEETINGS, COMMUNITY OF PRACTICE AND WEBINARS | | |
| - Does phone ownership increase empowerment among women? - How much do health workers and beneficiaries spend to access money digitally? - How much time is saved when using digital payment compared to cash. - What are the tangible key benefits and compelling value proposition to the users (health care and voluntary workers) of digitizing their payments and is there evidence to back up the benefits? - Beyond improving efficiencies for organization, do field recipients find it preferred than cash payments. - Evaluate the policy frameworks and regulatory environments around digital payments. - Describe the monitoring systems for digital payments - Who is to monitor? - How do we boost the morale of implementers without cash? - What is the impact with the morale of the frontline workers and other stakeholders? - What are the causes for non-implementation of digital health payments? - How can we enhance digital payment in remote areas with power networks? - What are the challenges experienced by Mobile Network Operators (MNOs) in the provision of digital health payments services? - Governments use digital systems majorly for emergencies and health campaigns but not routinely for non- salaried health workers unlike NGOs where its done routinely. - Limited financial literacy undermines trust such that users are easily affected by scams and fraud where there is limited understanding of products and risks. - Lack of an effective complaint mechanism with unclear and complex processes. - Organizational (in)efficiencies and models for improving efficiencies, how do we make digital payments instant? - Evidence on the impact of digital payment systems on program outcomes, efficiency and delivery in Nigeria. - No evidence on the depth, penetration, geographical reach and technological level of digital infrastructure in Nigeria. - Digital payment recipients’ perspectives including satisfaction, challenges, preferences were not documented in literature to the best of our knowledge. - Studies that provide evidence on this link are desirable for enacting policies that will support digital payment systems for health workers in Nigeria. - How to link the various systems already in place to support digitation of payments? - Mixed picture on cost: Savings from transport, corruption Vs taxes and charges – How do we optimize gains without shifting the burden of transactional costs to payees? - The need for a legal framework that supports the digital payment system - Compatibility of the digital payment system with the existing payment systems at the district or national level - Measuring the association between digitizing and program outcomes - Linking digitization with health system performance – indications of inefficiencies, we need to know the extent - Determine the extent of payment delays, incomplete payments, and the time taken to rectify, implications for; Timeliness; Feedback loops; Health worker performance and morale - Verification of work performed, and payment made: how do you achieve this and maintain timeliness? What alternatives can be put in place? | | |
