# Supplementary material for: A research agenda for digital payments of health workers in large-scale health campaigns in sub-Saharan Africa
Source: BMJ Glob Health. 2026 Feb 15;10(Suppl 4):e017476. doi: 10.1136/bmjgh-2024-017476 (PMC12962003; doi:10.1136/bmjgh-2024-017476)
Supplement: online supplemental table 2 [file bmjgh-10-Suppl_4-s002.docx]

# SUPPLEMENTARY TABLE 2 - RESEARCH PRIORITY SCORES AND RANKS AFTER APPLICATION OF CHNRI

| *RESEARCH OPTIONS* | *RANK* | *AEA (%)* | *RPS*  *(%)* | *Sustaina-bility and Equity:* | *Answera-bility and Ethics* | *Effectiv*  *eness* | *Impact* | *Method-ological Rigor* |
| --- | --- | --- | --- | --- | --- | --- | --- | --- |
| What are the minimum requirements for health systems to digitize payments responsibly? | 1 | 82.68 | 38.60 | 0.85 | 0.81 | 0.84 | 0.81 | 0.82 |
| How can digital payments be optimized to enhance the effectiveness of large-scale health campaigns in SSA? | 2 | 81.86 | 36.75 | 0.81 | 0.81 | 0.83 | 0.82 | 0.83 |
| What incentives should accompany the process of adopting digital payment to encourage its take-up by healthcare sector players? | 3 | 81.68 | 36.32 | 0.84 | 0.79 | 0.81 | 0.83 | 0.81 |
| What is the cost and benefit of implementing DPS compared to Cash, in health campaigns (e.g. in the number of days worked)? | 4 | 81.60 | 36.16 | 0.81 | 0.81 | 0.84 | 0.81 | 0.81 |
| What is the coverage of mobile money agents in different administrative units and how does this affect the uptake and satisfaction with digital payments for health campaigns? | 5 | 80.58 | 33.93 | 0.81 | 0.80 | 0.83 | 0.80 | 0.78 |
| What is the link between digital Financial Inclusion and the Economic Empowerment of Community HW in SSA? | 6 | 80.44 | 33.63 | 0.79 | 0.81 | 0.84 | 0.79 | 0.79 |
| What is the impact of digital payment of health workers on provider behavior (including efficiency, quality of care, cost reduction) and access to services (accessibility and cost reduction)? | 7 | 80.24 | 33.21 | 0.82 | 0.82 | 0.81 | 0.79 | 0.77 |
| How is personal data secured in digital payment systems and how can it be optimized? | 8 | 80.08 | 32.83 | 0.83 | 0.81 | 0.83 | 0.78 | 0.76 |
| What are the social inclusion and equity challenges in adopting digital payments? | 9 | 79.62 | 31.96 | 0.80 | 0.77 | 0.81 | 0.78 | 0.81 |
| How do digital payments reduce corruption tendencies? | 10 | 79.16 | 31.05 | 0.81 | 0.77 | 0.80 | 0.79 | 0.79 |
| What is the impact of digital payment (compared to cash), on improving health outcomes, campaign effectiveness and coverage and quality of vaccination? | 11 | 78.90 | 30.56 | 0.78 | 0.77 | 0.81 | 0.79 | 0.80 |
| How does digitizing performance-based incentives influence the motivation and retention of healthcare workers in health campaigns? | 12 | 78.80 | 30.37 | 0.78 | 0.78 | 0.79 | 0.79 | 0.80 |
| What proportion of healthcare workers own phones / mobile devices and or are registered on a digital payment account? | 13 | 78.80 | 30.36 | 0.81 | 0.79 | 0.78 | 0.77 | 0.80 |
| What is the role of AI in automating digital payment processes for health workers; and how can the negative implications be addressed? | 14 | 78.66 | 30.11 | 0.80 | 0.79 | 0.78 | 0.78 | 0.78 |
| How can Monitoring and Evaluation indicators; for digital payments, be integrated / reflected in routine health worker surveys? | 15 | 78.18 | 29.15 | 0.80 | 0.80 | 0.80 | 0.76 | 0.76 |
| How do DHP systems impact the motivation of health workers, and what strategies can be employed to ensure positive HW performance? | 16 | 78.14 | 29.11 | 0.78 | 0.80 | 0.77 | 0.77 | 0.79 |
| What are the barriers to, and facilitators experienced in the provision of digital payment for health workers? | 17 | 78.14 | 29.09 | 0.81 | 0.78 | 0.75 | 0.78 | 0.79 |
| How are DHP systems in SSA organized to foster financial inclusion of health workers participating in large-scale campaigns? | 18 | 78.04 | 28.92 | 0.76 | 0.77 | 0.80 | 0.78 | 0.80 |
| How do cultural/gender dynamics influence uptake of DHP for healthcare workers in SSA? | 19 | 77.96 | 28.74 | 0.77 | 0.77 | 0.82 | 0.77 | 0.78 |
| What strategies and technologies can be developed to ensure the interoperability of DHP systems on a global scale, facilitating seamless cross-border healthcare access and payment processing? | 20 | 77.94 | 28.72 | 0.77 | 0.77 | 0.77 | 0.77 | 0.82 |
| How can the efficiency of digital payment systems for health workers be optimized (i.e. reducing payment delays, reducing administrative costs, and foster timely accountability)? | 21 | 77.64 | 28.20 | 0.77 | 0.77 | 0.80 | 0.78 | 0.77 |
| What is the policy and regulatory environment for digital payments for health workers in SSA? | 22 | 77.64 | 28.16 | 0.79 | 0.78 | 0.76 | 0.74 | 0.81 |
| What, and how effective, are the existing strategies to address infrastructural challenges for digital payment? | 23 | 77.46 | 27.84 | 0.78 | 0.81 | 0.78 | 0.76 | 0.75 |
| What is the role of implementation science in accelerating use of digital health payment systems and processes? | 24 | 77.34 | 27.63 | 0.76 | 0.76 | 0.77 | 0.77 | 0.81 |
| What is the feasibility and effectiveness of integrating DHP systems in healthcare infrastructure in low-resource settings to improve healthcare access and quality while ensuring data security and equity? | 25 | 77.28 | 27.52 | 0.78 | 0.75 | 0.80 | 0.76 | 0.78 |
| How can we simplify the verification of payment of beneficiaries while guaranteeing timeliness, completeness, accuracy and security? | 26 | 76.56 | 26.23 | 0.78 | 0.75 | 0.73 | 0.76 | 0.80 |
| What is the impact of digital payment for health workers on telecom providers operating in areas where access to phone networks is limited? | 27 | 76.34 | 25.89 | 0.75 | 0.78 | 0.78 | 0.74 | 0.78 |
| What strategies can be employed to overcome resistance to change and promote the widespread adoption of DPS among health workers? | 28 | 76.28 | 25.82 | 0.76 | 0.76 | 0.78 | 0.77 | 0.76 |
| How can adoption, acceptability and scale up of digital payments in large scale health campaigns promote the financial inclusion of health workers? | 29 | 75.94 | 25.13 | 0.71 | 0.75 | 0.76 | 0.81 | 0.78 |
| How does performance-based evaluation of DHP systems fit in with the indicators, and processes of the current (status quo) payment system? | 30 | 74.60 | 23.04 | 0.71 | 0.74 | 0.79 | 0.74 | 0.74 |
| How can the process of registering health worker data be automated to enhance security and user trust through biometric authentication methods? | 31 | 74.48 | 22.89 | 0.74 | 0.77 | 0.76 | 0.73 | 0.73 |
| What is the landscape of the different digital payment platforms in the African region and how do these influence the uptake of digital payment? | 32 | 73.98 | 22.15 | 0.73 | 0.74 | 0.76 | 0.73 | 0.75 |
| What is the willingness of governments in SSA to scale up digital payment, and what efforts are in place to foster the transition to Digital Health payment? | 33 | 71.44 | 18.60 | 0.70 | 0.71 | 0.72 | 0.71 | 0.73 |
| What are the perceptions of key digital health payment stakeholders in the ecosystem about digital health payments? | 34 | 67.72 | 12.00 | 0.74 | 0.76 | 0.77 | 0.37 | 0.75 |
| How can access to digital payments be increased among vulnerable health workers (the marginalized [persons with disabilities, the elderly, illiterate women], Living in hard-to-reach areas, and those living in areas with limited mobile technologies) and how can they be designed to ensure security? | 35 | 67.18 | 6.50 | 0.79 | 0.16 | 0.84 | 0.78 | 0.79 |
