# Supplementary material for: A research agenda for digital payments of health workers in large-scale health campaigns in sub-Saharan Africa
Source: BMJ Glob Health. 2026 Feb 15;10(Suppl 4):e017476. doi: 10.1136/bmjgh-2024-017476 (PMC12962003; doi:10.1136/bmjgh-2024-017476)
Supplement: online supplemental table 3 [file bmjgh-10-Suppl_4-s003.docx]

SUPPLEMENTARY TABLE 3 – THE TOP 15 RESEARCH OPTIONS, RESEARCH PRIORITY SCORES AND RANKS AFTER APPLICATION OF CHNRI METHODOLOGY

| **RESEARCH OPTIONS** | **RANK** | **RPS (%)** |
| --- | --- | --- |
| What are the minimum requirements for health systems to digitize payments responsibly? | **1** | **38.60** |
| How can digital payments be optimized to enhance the effectiveness of large-scale health campaigns in SSA? | **2** | **36.75** |
| What incentives should accompany the process of adopting digital payment to encourage its take-up by healthcare sector players? | **3** | **36.32** |
| What is the cost and benefit of implementing DPS compared to Cash, in health campaigns (e.g. in the number of days worked)? | **4** | **36.16** |
| What is the coverage of mobile money agents in different administrative units and how does this affect the uptake and satisfaction with digital payments for health campaigns? | **5** | **33.93** |
| What is the link between digital Financial Inclusion and the Economic Empowerment of Community HW in SSA? | **6** | **33.63** |
| What is the impact of digital payment of health workers on provider behavior (including efficiency, quality of care, cost reduction) and access to services (accessibility and cost reduction)? | **7** | **33.21** |
| How is personal data secured in digital payment systems and how can it be optimized? | **8** | **32.83** |
| What are the social inclusion and equity challenges in adopting digital payments? | **9** | **31.96** |
| How do digital payments reduce corruption tendencies? | **10** | **31.05** |
| What is the impact of digital payment (compared to cash), on improving health outcomes, campaign effectiveness and coverage and quality of vaccination? | **11** | **30.56** |
| How does digitizing performance-based incentives influence the motivation and retention of healthcare workers in health campaigns? | **12** | **30.37** |
| What proportion of healthcare workers own phones / mobile devices and or are registered on a digital payment account? | **13** | **30.36** |
| What is the role of AI in automating digital payment processes for health workers; and how can the negative implications be addressed? | **14** | **30.11** |
| How can Monitoring and Evaluation indicators; for digital payments be integrated / reflected in routine health worker surveys? | **15** | **29.15** |
