# Supplementary material for: A research agenda for digital payments of health workers in large-scale health campaigns in sub-Saharan Africa
Source: BMJ Glob Health. 2026 Feb 15;10(Suppl 4):e017476. doi: 10.1136/bmjgh-2024-017476 (PMC12962003; doi:10.1136/bmjgh-2024-017476)
Supplement: online supplemental table 4 [file bmjgh-10-Suppl_4-s004.docx]

SUPPLEMENTARY TABLE 4 - THE TOP FIVE QUESTIONS, AND THEIR RANKING FOR EACH THEME

| THEME | *RESEARCH OPTION / QUESTIONS* | *RPS* | *RANK POST CHNRI* |
| --- | --- | --- | --- |
| FINANCIAL INCLUSION AND ECONOMIC EMPOWERMENT | What is the link between digital Financial Inclusion and the Economic Empowerment of Community Health Workers in SSA? | 0.336 | 6 |
|  | What are the social inclusion and equity challenges in adopting digital payments? | 0.320 | 9 |
|  | How are digital payment systems in SSA organized to foster financial inclusion of health workers participating in large-scale campaigns? | 0.289 | 18 |
|  | What is the role of implementation science in accelerating use of digital health payment systems and processes? | 0.276 | 24 |
|  | How can access to digital payments be increased among vulnerable health workers (the marginalized [persons with disabilities, the elderly, illiterate women, etc.], Living in hard-to-reach areas, and those living in areas with limited mobile technologies) and how can they be designed to ensure security? | 0.065 | 35 |
| ADOPTION AND ACCEPTANCE | How do digital health payment systems impact the motivation of health workers, and what strategies can be employed to ensure positive health worker performance? | 0.291 | 16 |
|  | What is the feasibility and effectiveness of integrating digital health payment systems at scale into healthcare infrastructure in low-resource settings to improve healthcare access and quality while ensuring data security and equity?" | 0.275 | 25 |
|  | How can adoption, acceptability and scale up of digital payments in large scale health campaigns promote the financial inclusion of health workers? | 0.251 | 29 |
|  | What is the willingness of governments in SSA to scale up digital payment, and what efforts are in place to foster the transition to Digital Health payment? | 0.186 | 33 |
|  | What are the perceptions of key digital health payment stakeholders in the ecosystem about digital health payments? | 0.120 | 34 |
| EFFICIENCY AND EFFECTIVENESS OF DIGITAL PAYMENT PLATFORMS. (TIMELINESS, COMPLETENESS, TRANSPARENCY, ACCOUNTABILITY, COST-EFFECTIVENESS, CONSISTENCY) | How can digital payments be optimized to enhance the effectiveness of large scale health campaigns in SSA? | 0.368 | 2 |
|  | What incentives should accompany the process of adopting digital payment to encourage its take-up by healthcare sector players? | 0.363 | 3 |
|  | What is the cost and benefit of implementing digital payments compared to Cash, during health campaigns (e.g. increase / decrease in the number of days worked)? | 0.362 | 4 |
|  | What is the coverage of mobile money agents in different administrative units and how does this affect the uptake and satisfaction with digital payments for health campaigns? | 0.339 | 5 |
|  | How do digital payments reduce corruption tendencies? | 0.311 | 10 |
| DIGITAL PAYMENT PROCESSES (HEALTH WORKER DATABASES, BENEFICIARY VERIFICATION, AND DISBURSEMENT) | What are the minimum requirements for health systems to digitize payments responsibly? | 0.386 | *1* |
|  | What is the impact of digital payment of health workers on provider behavior (including efficiency, quality of care, cost reduction) and access to services (accessibility and cost reduction)? | 0.332 | *7* |
|  | How is personal data secured in digital payment systems and how can it be optimized? | 0.328 | *8* |
|  | What are the barriers to, and facilitators experienced in the provision of digital payment for health workers? | 0.291 | *17* |
|  | What is the policy and regulatory environment for digital payments for health workers in SSA? | 0.282 | *22* |
