# Supplementary material for: A research agenda for digital payments of health workers in large-scale health campaigns in sub-Saharan Africa
Source: BMJ Glob Health. 2026 Feb 15;10(Suppl 4):e017476. doi: 10.1136/bmjgh-2024-017476 (PMC12962003; doi:10.1136/bmjgh-2024-017476)
Supplement: online supplemental file 1 [file bmjgh-10-Suppl_4-s005.docx]

**SUPPLEMENT S1: BMJ GLOBAL HEALTH AUTHOR REFLEXIVITY CHECKLIST**

This research was conducted through a collaborative partnership between institutions in low- and middle-income countries (LMICs), primarily Uganda, Senegal, and Nigeria, and collaborators from high-income countries (HICs), including Sweden and the United States. The study was coordinated by Makerere University School of Public Health in Uganda and supported by regional research institutions and local implementing organizations across Sub-Saharan Africa. All the stages, from study conceptualization, tool development, data collection, analysis, to manuscript preparation, were co-led by authors from LMICs

| **MANUSCRIPT TITLE -** A GLOBAL RESEARCH AGENDA FOR DIGITAL PAYMENTS OF HEALTH WORKERS IN LARGE SCALE HEALTH CAMPAIGNS IN SUB-SAHARAN AFRICA (SSA); BMJGH 2024-017476 | | |
| --- | --- | --- |
|  |  |  |
| **CORRESPONDING AUTHOR -** PROFESSOR PETER WAISWA | | |
|  | **Yes** | **No** |
| 1. Does the authorship include individuals whose PRIMARY affiliation is in the country/ies where the research/program activity was done?  If no, please explain - **NA** | Yes[EF1] |  |
| 2. Is the leadership, contribution, and ownership of this work by individuals from this/these country/ies recognized within the authorship? | Yes |  |
| 3. Is the leadership, contribution, and ownership of this work by each author correctly represented in the author contributions statement describing each author’s role in the research or program and preparation of the manuscript? | Yes |  |
| 4. Does the manuscript describe how this research or program experience addresses local program and/or policy priorities? | Yes |  |
| 5. Have the authors shared what is reported in this manuscript with in-country stakeholders OR do the authors have a plan for how what is reported in this manuscript will be shared with in-country stakeholders?  The results from this manuscript have partly been disseminated in meetings with the technical Advisory and working groups at the Ministry of Health, Uganda National Expanded Programme of Immunization (UNEPI); as well as Academia, and in scientific conferences. Similarly, the findings have been presented to stakeholders, country actors and implementers in digital payments from Senegal, Uganda, Nigeria, Ghana, Ivory coast, and Burundi. We further plan to continue disseminating the findings in a wider global dissemination meeting after the manuscript has been published. | Yes |  |
| 6. Does the manuscript describe how individuals in this/these countries were involved in study or program design and/or implementation? | Yes |  |
| 7. Does the manuscript include acknowledgement of other individuals who contributed to the research, program, or other aspects of this manuscript, but who are not recognized as authors, as well as a brief description of these individuals’ respective contributions? | Yes |  |
